# The urinary RNA atlas of patients with chronic kidney disease

**DOI:** 10.1038/s41598-023-46555-5

**Published:** 2023-11-04

**Authors:** Li-Zhi Lv

**Affiliations:** Department of Nephrology, Jurong People Hospital, No. 66, Ersheng Road, Jurong, 212499 Jiangsu Province China

**Keywords:** Computational biology and bioinformatics, Biomarkers

## Abstract

Chronic kidney disease (CKD) represents a significant global health burden. Currently employed CKD biomarkers are influenced by various factors and lack accuracy in reflecting early-stage renal fibrosis severity. Consequently, there is an urgent need for the identification of early, noninvasive CKD biomarkers. Urine, easily collectible and kidney-derived, has demonstrated potential as a diagnostic source for various kidney diseases by leveraging its RNA content. To address this, we obtained RNA-seq data pertaining to urinary RNAs from both CKD patients and healthy controls via the Gene Expression Omnibus database (GEO). The DEseq2 software was utilized to identify differentially expressed RNAs (DE-RNAs). To evaluate the overall accuracy of these DE-RNAs in urine, we performed Receiver Operating Characteristic analysis (ROC). Selected urinary RNAs were subsequently validated using reverse-transcription quantitative real-time Polymerase Chain Reaction (qRT-PCR) in conjunction with ROC analysis. Computational and experimental analyses revealed significant increases in miR-542-5p, miR-33b-5p, miR-190a-3p, miR-507, and CSAG4 within the urine of CKD patients, exhibiting high AUC values. In conclusion, our findings suggest that urinary RNAs hold promise as diagnostic biomarkers for CKD.

## Introduction

Chronic kidney disease (CKD) presents as a progressive condition, starting with inflammation and leading to fibrosis. It has emerged as a significant global health burden, affecting approximately 10% of adults^1^. The rising prevalence of diabetes is further contributing to an increase in diabetic nephropathy patients, thereby amplifying the overall burden of CKD. The absence of early clinical signs prior to irreversible kidney dysfunction poses a primary challenge in effectively managing CKD patients^2^. Currently, serum creatinine, urea nitrogen, and protein are the most commonly used biomarkers for diagnosing CKD. However, these biomarkers are influenced by factors such as age, diet, and infection status, limiting their diagnostic accuracy. Additionally, they are inadequate in detecting early renal fibrosis, which is crucial for timely intervention. Renal biopsy provides a gold standard for precise renal pathological examination; however, it is invasive, discomforting, and associated with potential complications. Consequently, it cannot be routinely repeated or applied as a screening tool for large populations. Therefore, there exists an urgent need to identify early and non-invasive biomarkers for CKD that can enhance disease management and improve patient outcomes.

Urine, as a product excreted by the kidneys, holds potential as a valuable source for exploring noninvasive biomarkers. Within urine, various types of RNAs, including mRNA, lncRNA, circRNA, miRNA, piRNA, and tRNA, have been identified^3^. Due to the ease of urine collection, urinary RNAs have emerged as promising noninvasive biomarkers for detecting kidney diseases. Previous studies have demonstrated the diagnostic utility of urinary RNAs in conditions such as bladder cancer^4^, renal cancer^5^, and prostate cancer^6^. Nonetheless, there remains a critical research gap concerning whether RNAs present in urine can serve as biomarkers for diagnosing CKD.

Therefore, in this study, we aimed to investigate urinary RNAs from both individuals with CKD and healthy controls in order to identify potential RNA biomarkers for diagnosing CKD. Through computational analysis of RNA-sequencing data, we characterized a diverse range of RNA classes present in the urine, including mRNAs, lncRNAs, circRNAs, miRNAs, piRNAs, and tRNAs. Furthermore, our analysis revealed several specific urinary RNAs that exhibited promising diagnostic potential for CKD.

## Methods

### RNA-sequencing (RNA-seq) data of urinary RNAs from CKD and healthy controls in GEO database

RNA-seq data pertaining to urinary RNAs in relation to CKD and healthy controls were retrieved from the GEO database (http://www.ncbi.nlm.nih.gov/geo/). We selected RNA-seq datasets based on the following criteria: (a) a minimum sample size of 10 samples, and (b) examination of RNA expression specifically in urine. Datasets that did not provide relevant or useful data for our analysis were excluded. Eventually, two GEO datasets, namely GSE121978 (consisting of 80 urine samples derived from CKD patients) and GSE128359 (including 47 urine samples obtained from healthy controls), met our inclusion criteria. Both of these GEO datasets were generated using Illumina HiSeq 2000 technology.

### Analysis of RNA-seq data

The quality of the RNA-seq data was assessed using the FastQC software. To remove adapter sequences, the Cutadapt software (V1.9.3) was employed. For circRNA and mRNA analysis, Hisat2 software^7^ was utilized, utilizing clear reads along with the circBase database^8^. Regarding miRNA data analysis, we followed a workflow previously described^8^. Adapter sequences were trimmed using Cutadapt software (V1.9.3), discarding reads larger than 14 nucleotides. These processed reads were then mapped against precursor miRNA sequences retrieved from miRBase (Release 21) via the Shrimp algorithm. In the case of other small noncoding RNAs encompassing piRNAs and tsRNAs, the reads were aligned against the human genomic sequence hg38 (GRCh38) employing Bowtie2 v2.2.7^9^. The resulting read alignment files were used for quantifying the expression of ncRNA annotations derived from Gencode v24^10^ and DASHR database^11^.

### Identification of dysregulated RNAs (DE-RNAs)

To mitigate batch effects and unwanted variations in RNA counts within each sample of the two GEO datasets, the SVA package was employed. Subsequently, the DEseq2 software was utilized to identify differentially expressed RNAs (DE-RNAs). In our analysis, RNAs meeting the following criteria were considered differentially expressed: reads per million greater than 1, p-value less than 0.01, and the absolute value of log2 fold change (FC) higher than 1.

### Collection of urine samples and RNA isolation

Twenty biopsy-proven CKD patients and twenty healthy volunteers were recruited from Jurong People Hospital, and their whole stream early morning urine samples were collected. The demographic and clinical characteristics of the CKD patients and healthy volunteers are presented in Table 1. Healthy controls were defined as individuals without abnormalities identified through routine urinalysis and having normal renal function [estimated glomerular filtration rate (eGFR) > 90 ml/min/1.73 m2]. Subsequently, the urine samples were centrifuged at 3000 g for 30 min at 4 °C, and the supernatant was carefully collected and stored at − 80 °C. To extract RNA from the supernatant, the TRIzol Reagent (Ambion, Life Technologies) was employed following the manufacturer's protocol (Ambion, Life Technologies, USA). Furthermore, the concentration and purity of the extracted RNA were assessed using the absorbance ratio at 260/280 measured with a NanoDrop 2000 spectrophotometer (Thermo, USA).

**Table 1:** The clinical profile of CKD patients and healthy volunteers.

Characteristic	Clinical cohort (n = 40)	
	Patients (n = 20)	Healthy controls (n = 20)
Age (year)	59.3 ± 18.44	60.07 ± 17.26
Female (%)	50	50
Serum creatinine (µmol/l)	141.00 ± 501.25	68.55 ± 11.75
24 h urine protein (g)	93.62 ± 593.69	ND
eGFR (ml/min/1.73 m^2^)	82.29 ± 33.66	103.84 ± 9.92

### Real-time RT-qPCR

RT-PCR was performed using specific primers of miR-542-5p, miR-33b-5p, miR-190a-3p, miR-507 and CSAG4 (Table 2). After RT (50 °C, 30 min), hot start (95 °C, 15 min) and 40 cycles of PCR (95 °C, 1 min; 60 °C, 1 min; and 72 °C, 1 min) was executed.

**Table 2:** RT and qPCR primers of the miR-542-5p, miR-33b-5p, miR-190a-3p, miR-507 and CSAG4.

	RT primer	qPCR primer	
		F	R
miR-542-5p	GTCGTATCCAGTGCAGGGTCCGAGGTATTCGCACTGGATACGACTCTCGT	CGTCGGGGATCATCATGTC	AGTGCAGGGTCCGAGGTATT
miR-33b-5p	GTCGTATCCAGTGCAGGGTCCGAGGTATTCGCACTGGATACGACGCAATG	CGCGGTGCATTGCTGTTG	AGTGCAGGGTCCGAGGTATT
miR-190a-3p	GTCGTATCCAGTGCAGGGTCCGAGGTATTCGCACTGGATACGACAGGAAT	CGCGCGCTATATATCAAACAT	AGTGCAGGGTCCGAGGTATT
miR-507	GTCGTATCCAGTGCAGGGTCCGAGGTATTCGCACTGGATACGACTTCACT	CGCGTTTTGCACCTTTTGG	AGTGCAGGGTCCGAGGTATT
CSAG4	TTAGGGAGAGCCTTTTGTTCCTGG	ATGTGGATGGGCCTCATCCAATTAG	TTAGGGAGAGCCTTTTGTTCCTGG

### Statistical analysis

SPSS 18.0 software was employed for statistical analysis, and GraphPad Prism 6.0 (GraphPad Software, San Diego, CA, USA) was utilized for graph generation. To assess the significance of differences in urinary RNA expression between groups, the Mann–Whitney *U* test was applied. A *p*-value less than 0.05 was considered statistically significant.

### Ethics approval

This study was approved by the Ethics Committee of Jurong People Hospital. Informed consent was waived by the Ethics Committee of Jurong People Hospital. All the experiments were performed in accordance with the approved guidelines and complied with the Declaration of Helsinki. The urine samples were collected from the residual urine after routine urine test, which belongs to the exemption of informed consent. Therefore, the participants didn’t provide written informed consent.

## Results

### Differential abundance analysis of urinary RNAs in CKD patients compared to healthy controls

**Figure 1:**
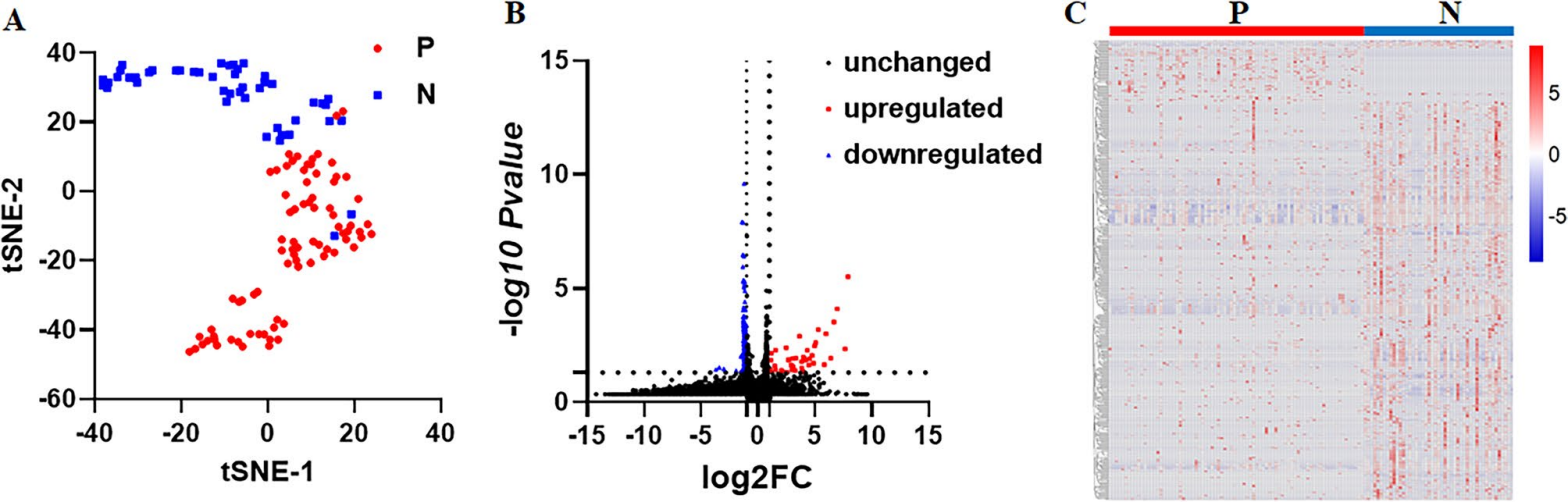
RNA expression in urine derived from CKD patients (P) and healthy controls (N). (**A**) tSNE analysis. (**B**) Volcano plot. (**C**) Heat map.

To explore the abundance of urinary RNAs, we performed an analysis using a dataset consisting of 80 CKD samples (GSE121978) and 47 healthy controls (GSE128359). This analysis encompassed a wide range of RNA types, including 36 circRNAs, 58,427 long RNAs (comprising mRNAs and lncRNAs), 1,636 miRNAs, 531 piRNAs, and 24 tRNAs (Table S1). Notably, the application of tSNE analysis revealed clear clustering of the CKD and healthy control samples based on their urinary RNA profiles (Fig. 1A). Furthermore, through visual examination of the volcano plot and heat map, distinct patterns of urinary RNA expression were observed, effectively differentiating CKD patients from healthy controls (Fig. 1B,C).

Using the DESeq2 software, we identified a total of 138 differentially expressed RNAs (DE-RNAs) meeting the significance criteria of |log2 fold change (FC)| ≥ 1 and a p-value ≤ 0.01. Out of these, 16 RNAs were found to be upregulated, while 122 were downregulated in CKD samples compared to healthy controls (Table S2).

### Selection of candidate urinary RNAs for the diagnosis of CKD

To assess the overall accuracy of 138 differentially expressed RNAs (DE-RNAs) in discriminating between various groups of CKD patients and healthy controls, ROC analysis was conducted. Specifically, these DE-RNAs were selected from a pool of urinary RNAs with reads above 1 per million. The analysis revealed that among the 100 RNAs under investigation, each exhibited distinct area under the curve (AUC) values, with the highest AUC (AUC = 0.85625) observed for hsa-miR-542-5p (Table S3).

To identify the optimal combination of diagnostic markers for CKD, further scrutiny was focused on RNAs with an AUC exceeding 0.75. As a result, six out of the 100 RNAs were shortlisted: miR-542-5p, miR-33b-5p, miR-190a-3p, miR-507, Chondrosarcoma-Associated Gene 2/3 Protein Pseudogene (CSAG4), and miR-95-5p (Table S3). These selected RNAs demonstrated promising diagnostic potential for CKD.

### Identification of the six urinary RNAs for the diagnosis of CKD

**Figure 2:**
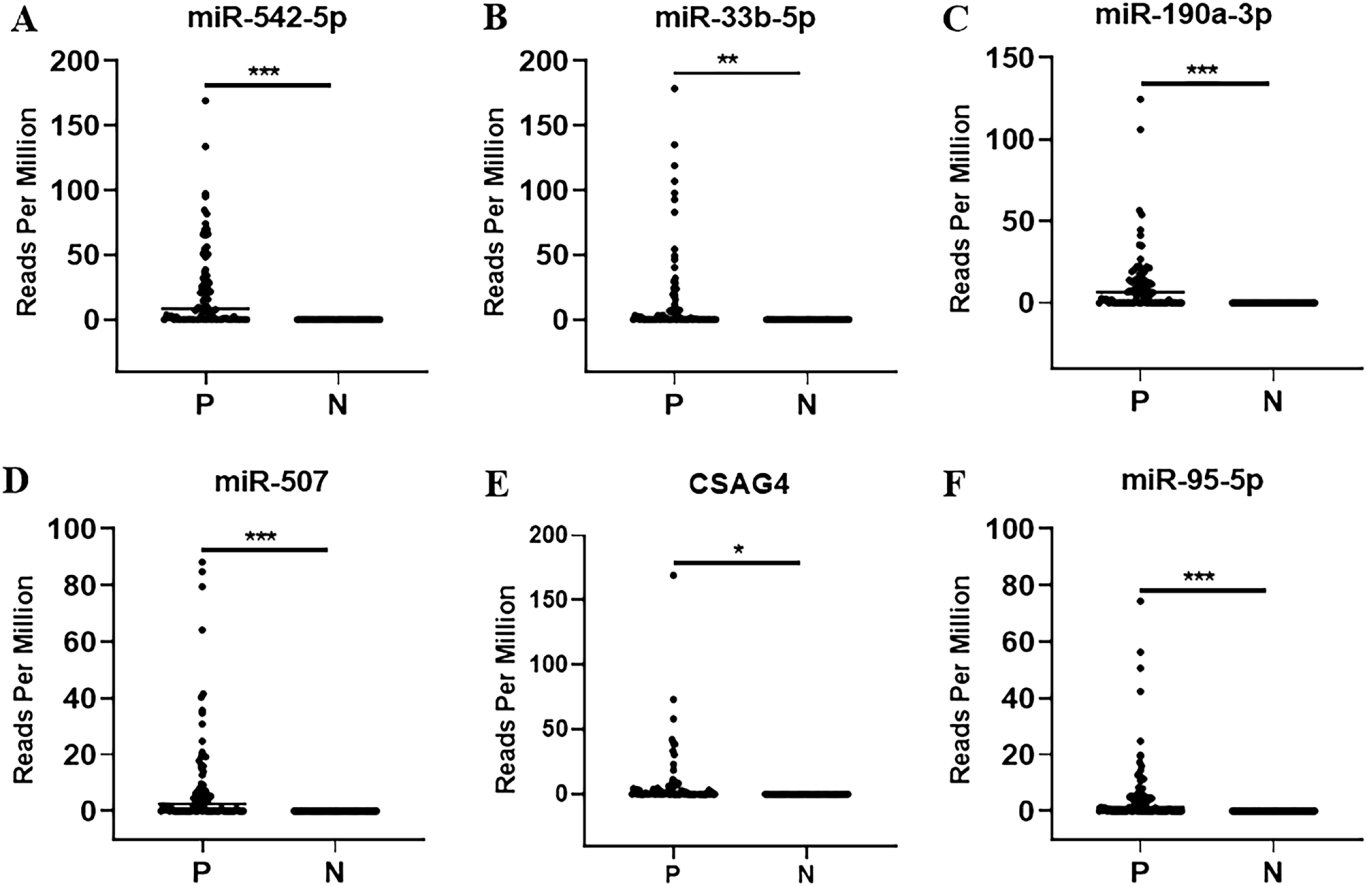
Urinary RNA expression levels of CKD patients (P) and healthy controls (N). (**A**–**F**) The expression levels of miR-542-5p, miR-33b-5p, miR-190a-3p, miR-507, CSAG4, and miR-95-5p in urine from CKD patients (N = 80) and healthy controls (N = 47) by RNA sequencing. Each value is the mean ± SD; *P < 0.05; **P < 0.01; ***P < 0.001.

**Figure 3:**
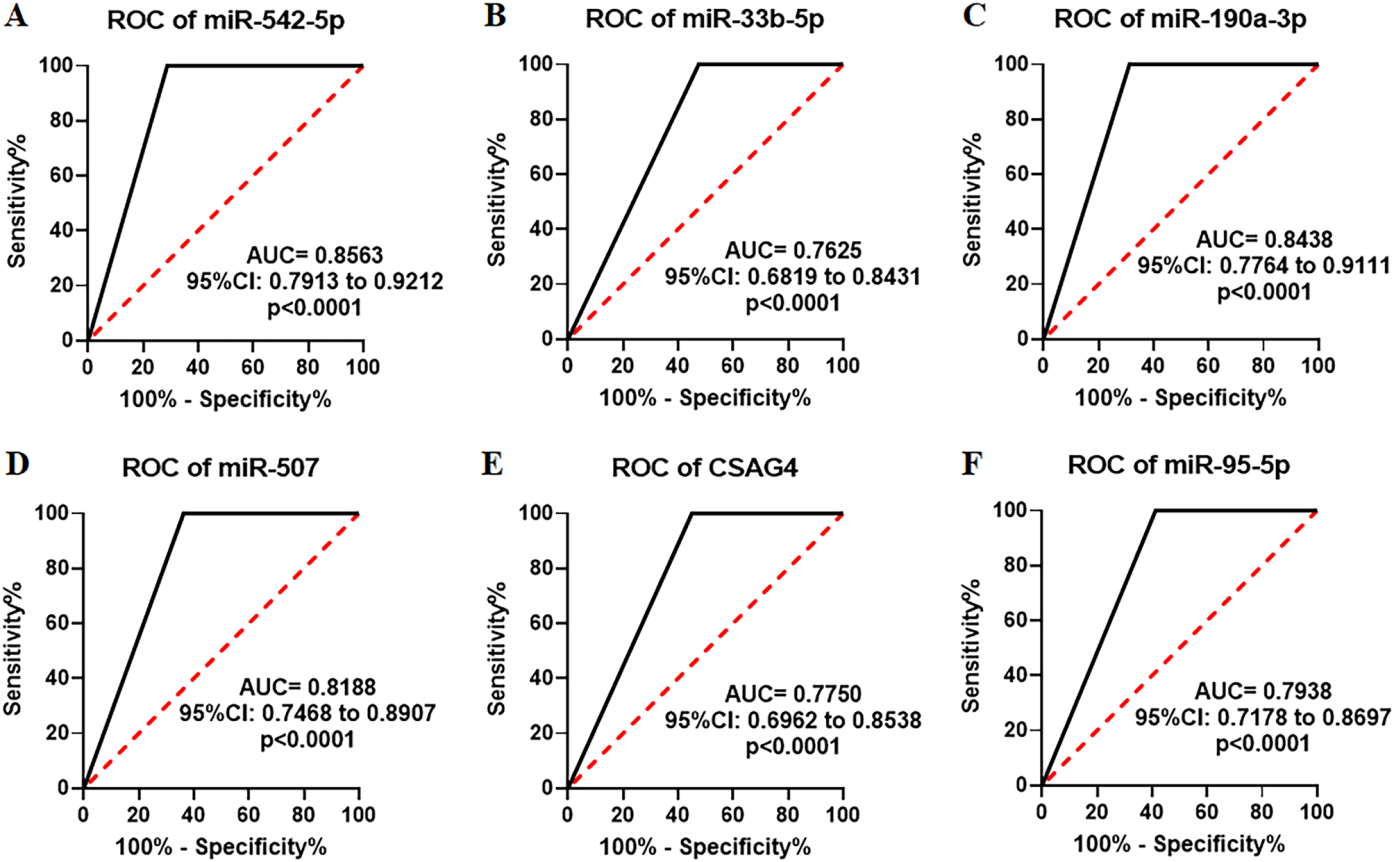
ROC curve analysis for the urinary RNAs of CKD patients (P) and healthy controls (N). (**A**–**F**) ROC curve of miR-542-5p, miR-33b-5p, miR-190a-3p, miR-507, CSAG4, and miR-95-5p in urine from CKD patients (N = 80) and healthy controls (N = 47) by RNA sequencing.

**Figure 4:**
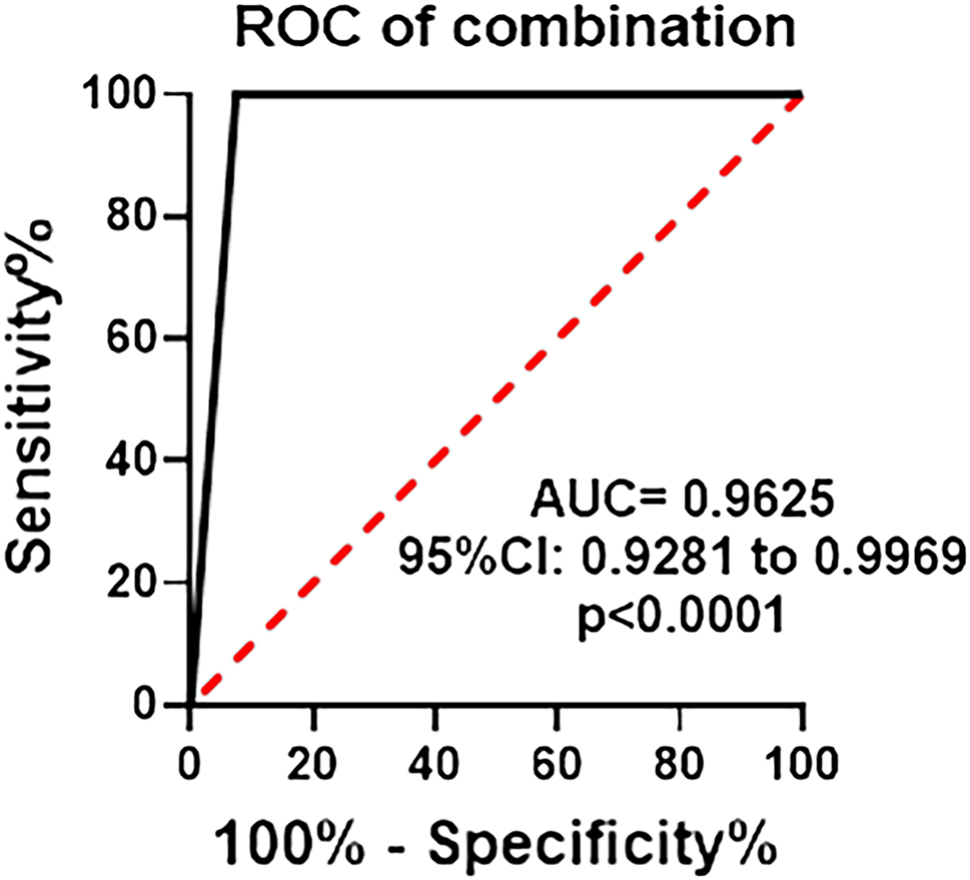
ROC curve analysis for the combined urinary RNAs of CKD patients (P) and healthy controls (N). ROC curve for the combination of miR-542-5p, miR-33b-5p, miR-190a-3p, miR-507 and CSAG4 in urine from CKD patients (N = 80) and healthy controls (N = 47) by RNA sequencing.

To validate the differential expression of the six candidate RNAs in urine, we conducted further analysis using the Mann–Whitney *U* test and ROC analysis. As shown in Fig. 2A–F, the expression levels of miR-542-5p, miR-33b-5p, miR-190a-3p, miR-507, CSAG4, and miR-95-5p were significantly elevated in CKD patients when compared to healthy controls (Fig. 2A–F). To assess the diagnostic value of these six urinary RNAs for CKD detection, the ROC curve analysis was subsequently performed. As shown in Fig. 3A–F, the AUC values of these six urinary RNAs were 0.8563 (95% CI 0.7913–0.9212) for miR-542-5p, 0.7625 (95% CI 0.6819–0.8431) for miR-33b-5p, 0.8438 (95% CI 0.7764–0.9111) for miR-190a-3p, 0.8188 (95% CI 0.7468–0.8907) for miR-507, 0.7750 (95% CI 0.6962–0.8538) for CSAG4, and 0.7938 (95% CI 0.7178–0.8697) for miR-95-5p (Fig. 3A–F). Then, the diagnostic value of the combination of these urinary RNAs was evaluated by a logistic regression model. Remarkably, the combination of miR-542-5p, miR-33b-5p, miR-190a-3p, miR-507, and CSAG4 yielded the highest AUC of 0.9625 (95% CI 0.9281–0.9969) (Fig. 4). Moreover, the sensitivity and specificity of the combination were 100% and 92.50%, respectively. Based on the outcomes of the aforementioned logistic regression, we computed the risk score (Logit(p)) based on the combination of miR-542-5p, miR-33b-5p, miR-190a-3p, miR-507, and CSAG4 (Table 3):

$$ {\text{Logit}}\left( p \right) = 0.{25}0*\left[ {{\text{miR-542-5p}}} \right] + {2}.{53}0*\left[ {{\text{miR-33b-5p}}} \right] + {2}.{291}*\left[ {{\text{miR-190a-30}}} \right] + {5}.0{52}*\left[ {{\text{miR-507}}} \right] + {43}.{813}*\left[ {{\text{CSAG4}}} \right] - {2}.0{58}. $$

**Table 3:** The risk score of the combination of the six urinary RNAs.

	B	S.E.	Wald	df	Sig	Exp(B)
miR-542-5p	0.2496	16.647	0.0002	1	0.9880	1.28363
miR-33b-5p	2.5298	163.83	0.0002	1	0.98768	12.5518
miR-190a-3p	2.2907	441.28	2.6E−05	1	0.9958	9.88197
miR-507	5.0519	538.73	8.7E−05	1	0.9925	156.322
CSAG4	43.812	700.59	0.0039	1	0.9501	1.1E+19
Constant	− 2.058	0.4335	22.543	1	2.1E−06	0.12766

The obtained results strongly suggest that the combination of miR-542-5p, miR-33b-5p, miR-190a-3p, miR-507, and CSAG4 in urine serves as an ideal biomarker for the detection of CKD.

### Validation the six urinary RNAs for the diagnosis of CKD

**Figure 5:**
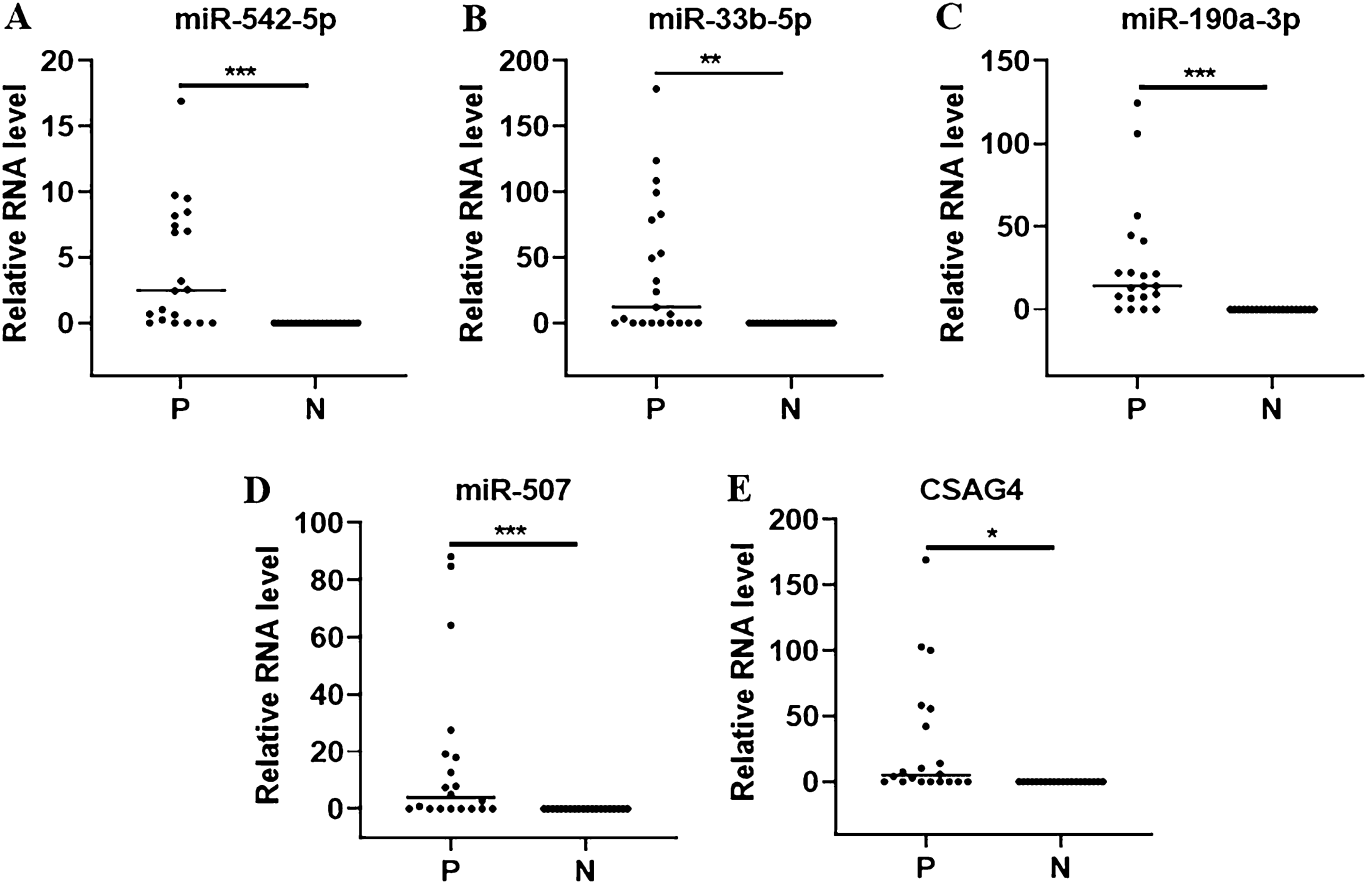
The expression levels of urinary RNA of the CKD patients (P) and healthy controls (N) by qRT-PCR. (**A**–**F**) The expression levels of miR-542-5p, miR-33b-5p, miR-190a-3p, miR-507 and CSAG4 in urine from CKD patients (N = 20) and healthy controls (N = 20) by qRT-PCR. Each value is the mean ± SD; *P < 0.05; **P < 0.01; ***P < 0.001.

**Figure 6:**
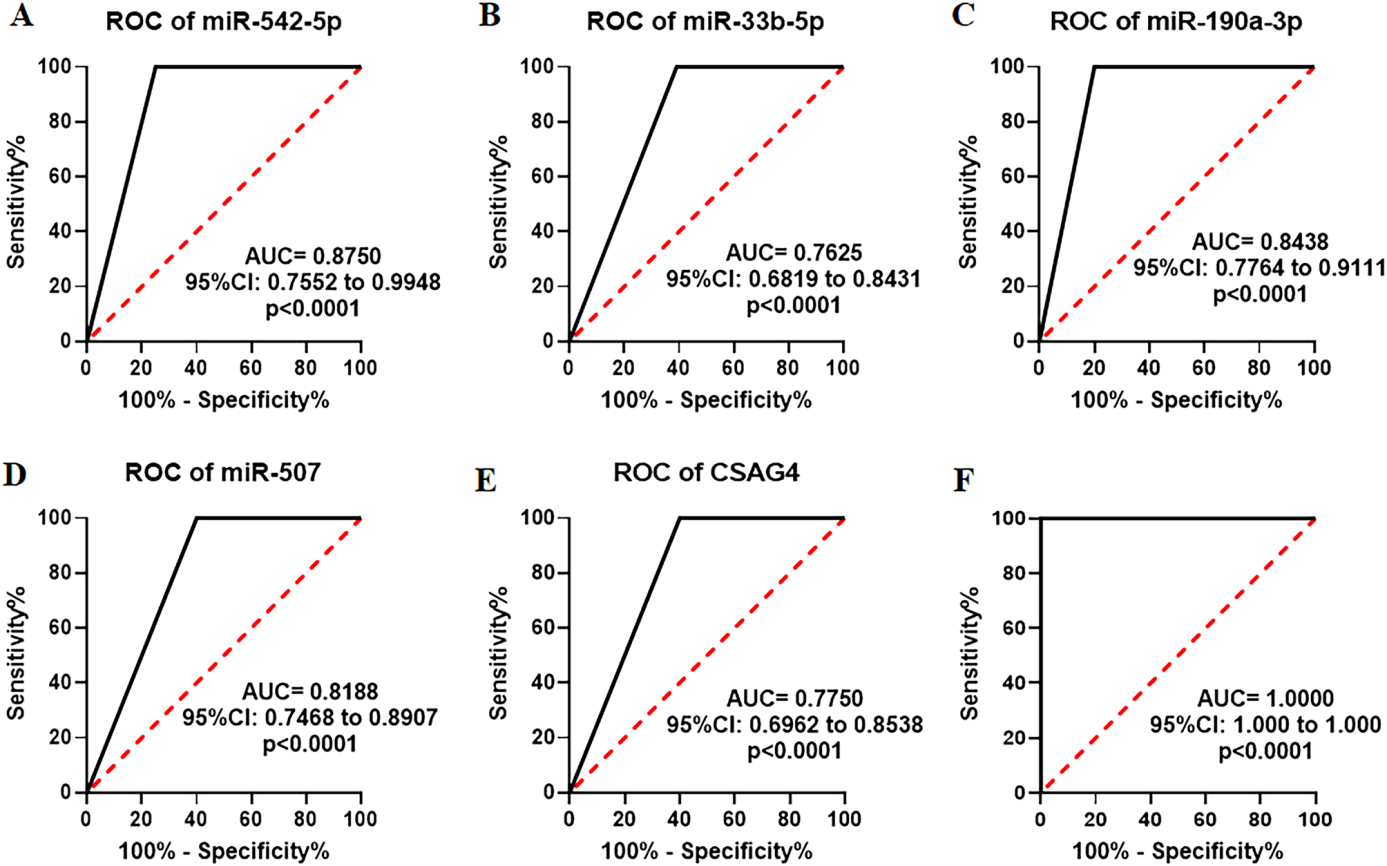
The ROC curve analysis for the urinary RNAs of CKD patients (P) and healthy controls (N). (**A**–**E**) ROC curve of miR-542-5p, miR-33b-5p, miR-190a-3p, miR-507 and CSAG4 in urine from CKD patients (N = 20) and healthy controls (N = 20) by qRT-PCR. (**F**) ROC curve for the combination of miR-542-5p, miR-33b-5p, miR-190a-3p, miR-507 and CSAG4 in urine from CKD patients (N = 20) and healthy controls (N = 20) by qRT-PCR.

A total of 40 urine samples were collected, consisting of 20 CKD patients and 20 healthy volunteers. The expression levels of miR-542-5p, miR-33b-5p, miR-190a-3p, miR-507, and CSAG4 in the urine were measured using RT-qPCR. Consistent with the findings from previous RNA sequencing results, the expression levels of miR-542-5p, miR-33b-5p, miR-190a-3p, miR-507, and CSAG4 in the urine of CKD patients were significantly up-regulated (Fig. 5). Subsequently, ROC curve analysis was conducted to assess the diagnostic value of urinary RNAs in the diagnosis of CKD, and the results revealed that urinary miR-542-5p, miR-33b-5p, miR-190a-3p, miR-507, and CSAG4 exhibited an AUC of *0.8750 (95% CI*, *0.7552–0.9948*, *p* < *0.001)*, *0.7625 (95% CI*, *0.6819–0.8431*, *p* < *0.001)*, *0.8438 (95% CI*, *0.7764–0.9111*, *p* < *0.001)*, *0.8188 (95% CI*, *0.7468–0.8907*, *p* < *0.001)* and *0.7750 (95% CI*, *0.6962–0.8538*, *p* < *0.001*), respectively (Fig. 6A–E). The AUC of the combination of miR-542-5p, miR-33b-5p, miR-190a-3p, miR-507, and CSAG4 was 1.0000 (95% CI 1.0000–1.0000) (Fig. 6F). Thus, urinary miR-542-5p, miR-33b-5p, miR-190a-3p, miR-507, and CSAG4 could serve as ideal diagnostic biomarker for CKD.

## Discussion

Until now, the diagnosis of CKD has primarily relied on urinary protein analysis and assessing changes in the glomerular filtration rate. Diagnostic biomarkers at the gene transcription level, widely employed in the diagnosis of tumors and various diseases, have not been extensively utilized for CKD due to the invasive nature of kidney biopsy. However, as growing evidence indicates the presence of RNAs in urine, similar to serum/plasma samples, the analysis of urinary RNAs presents a new avenue to gain insights into CKD. This non-invasive approach offers a promising opportunity to explore and understand CKD in a novel way.

**Figure 7:**
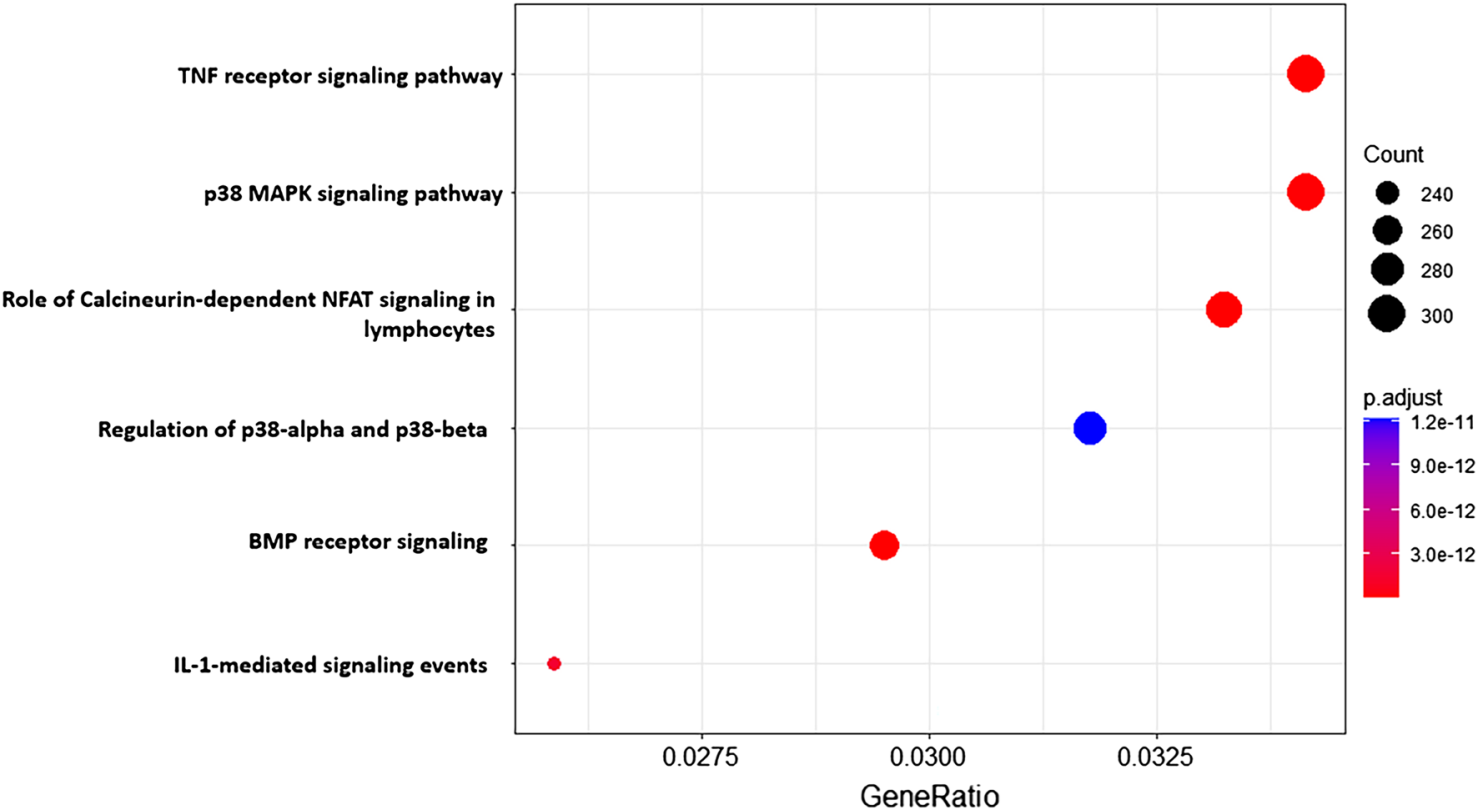
The biological pathways of miR-542-5p, miR-33b-5p, miR-190a-3p, miR-507, CSAG4, and miR-95-5p.

In this study, high-throughput RNA-sequencing was employed to analyze urinary RNAs with the aim of identifying potential biomarkers for CKD diagnosis. Urine, being easily collectible, has been regarded as an ideal source for kidney-related disease biomarkers. Our analysis revealed the presence of diverse RNA classes in urine, including mRNA, lncRNA, circRNA, miRNA, piRNA, and tRNA. Notably, miR-542-5p, miR-33b-5p, miR-190a-3p, miR-507, and CSAG4 in urine emerged as promising biomarkers for CKD. Both plasmatic transrenal and postrenal cell-free RNA were identified in urine; however, the focus of existing studies has predominantly been on RNA released into urine postrenally through mechanisms such as apoptosis, necrosis, and active secretion. Previous investigations have successfully detected survirin, cytokeratin 20, mucin 7, and Ki-67 mRNAs in the urine of patients with bladder cancer and various urinary tract infections, highlighting the cells of the urogenital tract as major contributors of urinary RNAs^12^. Functional analysis of the identified miR-542-5p, miR-33b-5p, miR-190a-3p, miR-507, and CSAG4 biomarkers revealed their involvement in signal pathways relevant to renal damage, such as TNF receptor signaling pathway and p38 MAPK signaling pathway (Fig. 7). These findings suggest that miR-542-5p, miR-33b-5p, miR-190a-3p, miR-507, and CSAG4 might be actively or passively released by the kidneys during the progression of CKD. However, further research is necessary to elucidate the exact release mechanisms of these five indicators by the kidneys.

The detection and identification of RNAs in urine can be accomplished using various techniques such as RNA sequencing, microarray technologies, or reverse-transcription quantitative real-time PCR (qRT-PCR). In our study, we utilized RNA sequencing to identify dysregulated RNAs in the urine of CKD patients. However, for practical clinical application, RNA sequencing poses challenges when applied to urine samples. Currently, qRT-PCR is the most commonly used and convenient method for RNA detection. Therefore, further extensive studies are necessary to establish a qRT-PCR-based detection method for miR-542-5p, miR-33b-5p, miR-190a-3p, miR-507, and CSAG4 in urine before they can be effectively utilized in clinical practice.

## Conclusions

In conclusion, our study identified five urinary RNAs, specifically miR-542-5p, miR-33b-5p, miR-190a-3p, miR-507, and CSAG4, which exhibited significantly higher expression levels in CKD patients compared to healthy controls. These findings suggest that these five urinary RNAs hold great potential as diagnostic biomarkers for CKD.

### Supplementary Information

Supplementary Legends.

Supplementary Table S1.

Supplementary Table S2.

Supplementary Table S3.

## Data Availability

All data generated or analyzed during this study are included in this published article. Raw and processed data are available upon request from Li-Zhi Lv (844713944@qq.com).
