# Supplementary material for: The urinary RNA atlas of patients with chronic kidney disease
Source: Sci Rep. 2023 Nov 4;13:19084. doi: 10.1038/s41598-023-46555-5 (PMC10625525; doi:10.1038/s41598-023-46555-5)
Supplement: Supplementary file 1 — Supplementary Legends. [file 41598_2023_46555_MOESM1_ESM.docx]

**Urinary RNA atlas of patients with chronic kidney disease**

Supplementary table

Table s1. urinary RNAs of CKD patients and healthy controls

Table s2. DE-RNAs in the urine of CKD patients and healthy controls

Table s3. ROC analysis of DE-RNAs in the urine
